# Structural basis of the activation of metabotropic glutamate receptor 3

**DOI:** 10.1038/s41422-022-00623-z

**Published:** 2022-03-02

**Authors:** Wei Fang, Fan Yang, Chanjuan Xu, Shenglong Ling, Li Lin, Yingxin Zhou, Wenjing Sun, Xiaomei Wang, Peng Liu, Philippe Rondard, Pan Shi, Jean-Philippe Pin, Changlin Tian, Jianfeng Liu

**Affiliations:** 1 The First Affiliated Hospital of USTC, School of Life Sciences, Division of Life Sciences and Medicine, Joint Center for Biological Analytical Chemistry, Anhui Engineering Laboratory of Peptide Drug, Anhui Laboratory of Advanced Photonic Science and Technology, University of Science and Technology of China, Hefei, Anhui China; 2 Key Laboratory of Molecular Biophysics of MOE, International Research Center for Sensory Biology and Technology of MOST, College of Life Science and Technology, Huazhong University of Science and Technology (HUST), Wuhan, Hubei China; 3 Bioland Laboratory, Guangzhou Regenerative Medicine and Health Guangdong Laboratory, Guangzhou, Guangdong China; 4 Institut de Génomique Fonctionnelle (IGF), Université de Montpellier, CNRS, INSERM, Montpellier, France

**Keywords:** Cryoelectron microscopy, Molecular biology

Dear Editor,

Glutamate is used by most synapses in the brain and responsible for fast excitatory transmission, thus playing important roles in excitotoxicity and ammonium detoxification in the brain.^1^ Eight G protein-coupled metabotropic glutamate receptors (mGlus) are essential in sensing glutamate concentrations from the ten nanomolar to ten millimolar range in the brain.^2^ The mGlu3 is in high sequence homology with mGlu2, but mGlu3 is of greater interest because it is responsible for the detection of very low concentrations of glutamate.^2^ The mGlu3 is found in astrocytes and in both pre- and post-synaptic elements in neurons, whereas mGlu2 is mainly distributed in neurons, particularly in the preterminal region of axons, far from the active zone of neurotransmitter release.^3^ Accumulating evidence supports a role of mGlu3 not only in maintaining synaptic homeostasis but also in promoting neuronal and astrocyte survival in several pathological conditions.^4^ The mGlu3 has garnered attention as a potent therapeutic target for both psychiatric disorders and neurodegenerative diseases such as schizophrenia, Alzheimer’s disease, anxiety, depression, pain and addiction.^5,6^ Polymorphic variants of the gene encoding mGlu3 are linked to schizophrenia. Furthermore, recent studies have suggested that negative allosteric modulators (NAMs) of both mGlu3 and mGlu2 induced rapid antidepressant-like effects through related but divergent mechanisms of action.^7^ Moreover, the high sequence homology of mGlu3 and mGlu2 restricts the development of selective ligands, which demands the structures of mGlu3.

Here, we present three cryo-EM structures of human mGlu3 homodimer: the agonist-bound state (bound with LY2794193), the antagonist-bound state (bound with LY341495) and the antagonist/NAM-bound state (bound with LY341495 and VU0650786) at overall resolutions of 3.68 Å, 4.17 Å and 3.71 Å (Fig. 1a–c; Supplementary information, Figs. S2–S4, S5a–c). Human full-length mGlu3 was overexpressed and purified in the presence of the agonist LY2794193, or the antagonist LY341495 alone and/or with the NAM VU0650786 (Supplementary information, Fig. S1a–f). The function of the mGlu3 was evaluated by a G_i_ protein-based cAMP inhibition assay (Supplementary information, Fig. S1g, h). Each mGlu3 subunit is composed by a Venus flytrap (VFT) domain, a transmembrane domain (TMD) consisting of seven TM helices, and a cysteine-rich domain (CRD) connecting the VFT domain and the TMD. Local refinement of the VFT domains of the agonist- and antagonist/NAM-bound mGlu3 dimer was implemented, resulting in resolutions of 3.27 Å and 3.39 Å, respectively, which allow better illustration of the densities in the orthosteric site (Fig. 1d, e). The antagonist LY341495 interacted with residues T174, R64, R68 and K389 of Lobe 1 (LB1) and Y222 of Lobe 2 (LB2) of the VFT domain (Fig. 1d). The mGlu3-selective agonist LY2794193 shared a similar binding pocket with the antagonist, interacting with residues T174, R68, Q306 and K389 of LB1 and D301 of LB2 (Fig. 1e).

**Fig. 1:**
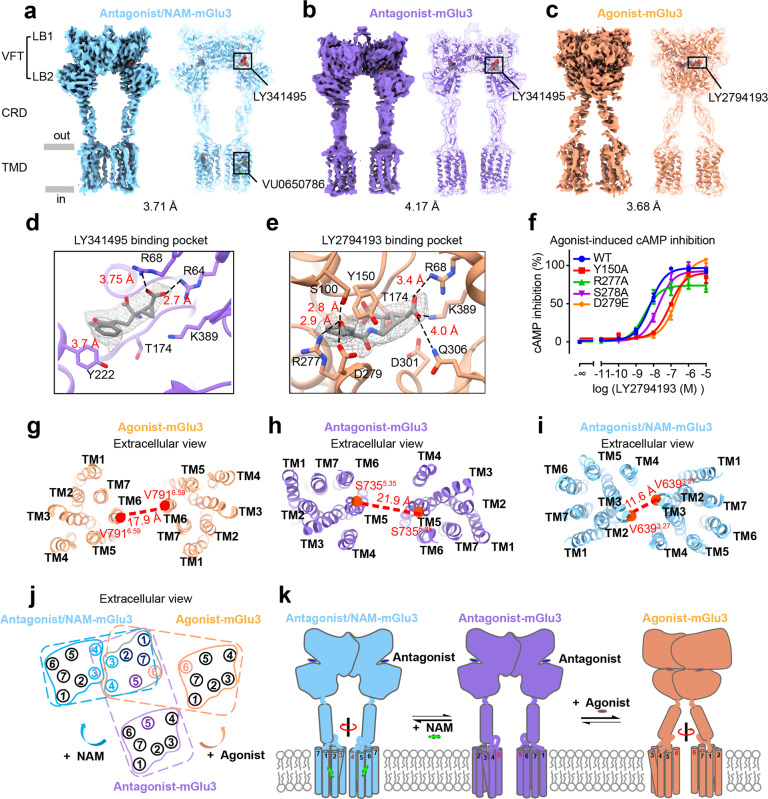
Cryo-EM structures of mGlu3 homodimer in agonist-, antagonist- and antagonist/NAM-bound states. **a**–**c** Cryo-EM maps and models of mGlu3 dimer in three different states. Antagonist/NAM–mGlu3, mGlu3 bound with the antagonist LY34149 and the NAM VU0650786 (**a**); antagonist–mGlu3, mGlu3 bound with the antagonist LY341495 (**b**); agonist–mGlu3, mGlu3 bound with the agonist LY2794193 (**c**). **d** Local density map of the antagonist LY341495 in the binding pocket. **e** Local density map of the agonist LY2794193 in the binding pocket. **f** LY2794193-induced cAMP inhibition in mGlu3 WT and mutants with substitutions in the ligand-binding pocket measured by the cAMP EPAC BRET sensor. Data are the means ± SEM from at least three independent experiments performed in technical triplicate. **g**–**i** Extracellular view of the TMDs of mGlu3 dimer in the agonist-bound state (**g**), antagonist-bound state (**h**) and antagonist/NAM-bound state (**i**). The red dotted lines indicate the distance between the two closest TM helices from two subunits. **j** Schematic diagram showing the extracellular view of mGlu3 TMD orientations in the antagonist/NAM-, antagonist- and agonist-bound states. The arrows indicate the movement of inactive-state mGlu3 upon agonist binding or NAM binding. **k** Antagonist-bound mGlu3 represents an inactive state, with the VFT domains in an open conformation and the TMDs farthest apart from each other (middle). Agonist binding in the cleft of the VFT lobes causes VFT domain closure and CRD rotation (right). Further binding of the NAM to the inactive mGlu3 shows little effect on the VFT domains but causes TMD rotation bringing the two TMDs closer (left). The TMD interface changed from TM5–TM5 to TM3/4–TM3/4, further stabilizing the receptor in a fully inactive state.

The agonists for mGlu3 were investigated for their potential in the treatment of anxiety and drug addiction. Among them, LY2794193 is a derivative of a non-selective agonist LY354740 which was reported to activate both mGlu2 and mGlu3.^8^ In the agonist-binding pocket of mGlu3, the extra m-methoxyphenyl ring of LY2794193 forms a π–π interaction with Y150, a cation–π interaction with R277, and H-bonds with S100 and D279; D279 corresponds to E273 of mGlu2 (Fig. 1f; Supplementary information, Fig. S6a, b). The mutation of D279 in mGlu3 to glutamic acid decreased the effect of LY2794193 by a 15-fold shift in EC_50_, while the mutation of E273 in mGlu2 to aspartic acid increased the affinity of LY2794193 by 10 fold, indicating the important role of this residue for the specificity of LY2794193 binding to mGlu3 versus mGlu2 (Fig. 1f; Supplementary information, Fig. S6b).

The activation of mGlu3 led to compaction of the dimer. The distance of LB2 in the VFT domain between the two subunits was 33.5 Å in the antagonist-bound state and decreased to 22.5 Å in the agonist-bound state. The distance of the CRDs at V526 between the two subunits decreased from 37.0 Å to 15.2 Å (Supplementary information, Fig. S7a, b). This compaction between CRDs is similarly observed in the structures of mGlu2,^9,10^ mGlu5,^11^ GABA_B_^12,13^ and CaSR^14^ during inactive-to-active transition, and in mGlu1^15^ upon switching from apo to the intermediate active state (Supplementary information, Fig. S9). Conformational changes in the VFT domain were then propagated to the TMD through the CRD. The apex region of ECL2 probably forms ionic interactions with the CRD, which plays a crucial role in transmitting conformational changes from VFT to the TMD (Supplementary information, Fig. S8). Deletion of residues R723 and E724 impaired LY2794193-induced cAMP inhibition, and the double mutation R723L/E724L markedly abolished the effect of LY2794193. Mutation of residues E721–E724 to alanine or deletion of these residues also decreased cAMP inhibition by LY2794193 (Supplementary information, Fig. S8b), thus confirming the important role of ECL2.

Activation of mGlu3 leads to a rearrangement of TMD interface from TM5–TM5 to TM6–TM6. The most proximal distance between the TM6 helices was 17.9 Å, with Cα of V791^6.59^ being used as a reference, only 4 Å closer compared to the 21.9 Å measured in the antagonist-bound form between TM5 helices at S735^5.35^ (Fig. 1g, h). Among other reported class C GPCRs, the most proximal distance between the TM6 helices in the agonist/PAM-bound mGlu2,^9,10^ mGlu5,^11^ GABA_B_^12^ and CaSR^14^ were 10.0 Å, 6.0 Å, 8.0 Å and 9.7 Å, respectively, whereas the TMD distance of NAM-bound mGlu2,^10^ apo mGlu5,^11^ apo GABA_B_^12^ and L-Trp-bound CaSR^14^ were 10.0 Å, 20.8 Å, 14 Å and 17.4 Å, respectively (Supplementary information, Fig. S9). Our structures indicated that the TM5–TM5 interface in the antagonist-bound mGlu3 was switched to a TM6–TM6 interface in the agonist-bound state, similar to what was reported for other class C GPCRs. However, the two TM6 in agonist-bound mGlu3 remain further apart from each other, in contrast to those of other active class C receptor. In addition, the agonist-bound mGlu3 is different from agonist-bound mGlu1, although the TMDs from two subunits of agonist-bound mGlu1 are also far from each other with a distance of 15.5 Å^15^ (Supplementary information, Fig. S10). Only one VFT domain of mGlu1 is bound with the agonist,^15^ likely representing an intermediate state known to be partially active, whereas both VFT domains of mGlu3 are bound with the agonist. Our structural data clearly indicate a unique TMD rearrangement of mGlu3 upon agonist binding, which is different from those of other class C GPCRs.

In the structure of antagonist/NAM-bound mGlu3, the two TMDs of mGlu3 underwent a remarkable twist and brought TM3/TM4 helices of both subunits into close proximity. Additional density inside the allosteric pocket of each mGlu3 TMD may correspond to the NAM VU0650786 (Supplementary information, Fig. S5d, e). The NAM-binding mode in mGlu3 was examined by mutagenesis. The mutations W782^6.50^A and F652^3.40^A largely impaired the effect of VU0650786 through decreasing its potency (increasing its IC_50_) by more than 23 fold compared to the wild-type (Supplementary information, Fig. S5f and Table S2). Interestingly, the Y656^3.44^A and F744^5.47^A mutations increased the NAM sensitivity, probably by decreasing steric hindrance. Similar antagonist/NAM-binding pockets and TM3/4–TM3/4 dimer interface have previously been observed in NAM-bound structures of mGlu2,^9,10^ suggesting that mGlu3 and mGlu2 may share a similar NAM-binding mode.

Comparing the structure of antagonist/NAM-bound mGlu3 with that of antagonist-bound mGlu3 revealed that the inactive VFT regions of both structures showed open conformations. The extracellular tips of the TM4, TM5 and TM6 helices in antagonist/NAM-bound mGlu3 underwent outward shifting due to the binding of the NAM in the TMD **(**Supplementary information, Fig. S11). Whereas the TMDs of antagonist-bound mGlu3 were separated, with two TM5 helices in closest proximity (Fig. 1h), the TMDs of antagonist/NAM-bound mGlu3 underwent a marked conformation twisting and entered into proximity to form a symmetric dimer interface composed of the TM3 and TM4 helices of each subunit (Fig. 1i). The proximate distance between the TM3 helices in the antagonist/NAM-bound mGlu3 was 11.6 Å (taking Cα of V639 as the reference), and the nearest distance between TM4 helices was 11.4 Å (taking Cα of Q688 as the reference) (Fig. 1i). The antagonist/NAM-bound mGlu3 with the TM3/4–TM3/4 interface in close proximity indicated a fully inactive state of mGlu3. Functional studies also showed that NAM further decreases cAMP inhibition compared to the antagonist alone, confirming that NAM stabilizes the fully inactivate state of mGlu3, likely acting as an inverse agonist (Supplementary information, Fig. S1h). Meanwhile, differences in the CRD–TMD angles were observed, which lead to slightly different orientations of the TMDs in the antagonist/NAM-bound mGlu3 versus antagonist/NAM-bound mGlu2 (PDB 7MTQ) (Supplementary information, Fig. S12).

Aligning a single TMD of the three different mGlu3 structures revealed the configuration transition of TMD dimers upon ligand binding (Fig. 1j). In the antagonist-bound state, the antagonist stabilizes mGlu3 in a conformation where the TM5 helices being the most proximal between two TMDs. Upon agonist binding, the VFT domains and CRDs of mGlu3 underwent conformational changes which were further propagated to the TMDs, rotating the TMDs anticlockwise (extracellular view) along the C2 symmetry axis, bringing TM6 helices facing each other. However, upon the further binding of NAM, the receptor displayed conformational changes opposite to those upon agonist binding. The TMDs underwent a clockwise rotation (extracellular view), which brought TM3/TM4 helices of two subunits into closer proximity, forming a TM3/4–TM3/4 dimer interface, with the TM6 helices located furthest away.

In summary, we report three cryo-EM structures of mGlu3 in agonist-, antagonist- and NAM/antagonist-bound states, respectively. Combined with functional assays, these structures enabled us to propose a structural basis for mGlu3 activation (Fig. 1k). The compaction between the VFT domains and CRDs of two subunits, and an anticlockwise twist of the TMDs from TM5–TM5 in the antagonist-bound state to TM6–TM6 in the agonist-bound state, were observed in mGlu3. However, the two TMDs of mGlu3 were observed to be far apart in the agonist-bound conformation, which is distinct from other mGlus in active state. This suggests that a direct contact between TM6 is not a prerequisite for mGlu-dependent activation of G proteins. Upon NAM binding, the TMDs undergo a clockwise twist, which results in a closer distance between TM3/TM4 helices from the two subunits, similar as that observed in the antagonist/NAM-bound mGlu2, suggesting a new interface for fully inactive state in mGlu dimers. Taken together, our data reveal a unique structural framework of mGlu3 activation and inactivation. Structural analysis of agonist-, antagonist- and antagonist/NAM-bound mGlu3 will promote future design and development of more efficient and accurate orthosteric or allosteric modulators for mGlu3.

## Supplementary information

Supplementary Information
