# Supplementary material for: Structural basis of the activation of metabotropic glutamate receptor 3
Source: Cell Res. 2022 Mar 2;32(7):695–8. doi: 10.1038/s41422-022-00623-z (PMC9253128; doi:10.1038/s41422-022-00623-z)
Supplement: Supplementary file 1 — Supplementary Information [file 41422_2022_623_MOESM1_ESM.pdf]

Supplementary Materials for

Structural basis of the activation of metabotropic glutamate receptor 3

Materials and Methods

Protein expression and purification

Human full-length mGlu3 (UniProt: Q14832) was optimized and synthesized by GenScript and then cloned into the pFastBac1 and pcDNA3.1 vectors. To facilitate expression and purification, the original mGlu3 signal sequence (1-22aa) was substituted by haemagglutinin (HA) signal sequence, followed by a flag epitope and a TEV cleavage site. Bac-to-Bac Baculovirus Expression System (Invitrogen) was used to generate high-titer mGlu3 recombinant baculovirus. *Spodoptera frugiperda (Sf9)* cells (Invitrogen) were infected by viral stock at a density of 3.5×10^6 cells per ml. The transfected sf9 cells were grown at 27 °C for 48 h and then collected by centrifugation and stored at −80 °C until use.

Cells were thawed and suspended in 25 mM HEPES, pH 7.5, 150 mM NaCl with cocktail protease inhibitor, then solubilized with 1.5% (w/v) n-dodecyl-β-d-maltopyranoside (DDM, Anatrace), 0.3% (w/v) cholesteryl hemisuccinate (CHS, Sigma-Aldrich) for 2 h at 4 °C. After centrifugation at 170,000 × g for 35 min, the supernatant was collected and incubated with Anti-Flag G1 Affinity Gel (GenScript) for 1 h at 4 °C. Protein bound gel was washed with ten column volumes of 25 mM HEPES, pH7.5, 150 mM NaCl, 10 mM $MgCl_2$, 0.1% DDM, 0.02% CHS and eluted with of 25 mM HEPES, pH 7.5, 150 mM NaCl, 0.1% DDM, 0.02% CHS, 200 μg/ml

FLAG peptide. The eluted protein was concentrated by Amicon Ultra centrifugal filter (MWCO 50 kDa), and finally purified by size-exclusion chromatography (SEC) on a Superose 6 increase 10/300GL column (GE Healthcare) in SEC1 buffer (25 mM HEPES, pH7.5, 150 mM HEPES, 0.01% DDM, 0.002% CHS, 10 μM LY2794193 (MedChemExpress)) for the agonist-mGlu3 sample , in SEC2 buffer(25 mM HEPES, pH7.5, 150 mM HEPES, 0.01% DDM, 0.002% CHS, 10 μM LY341495 (MedChemExpress)) for antagonist-mGlu3 sample, or in SEC3 buffer (25mM HEPES, pH7.5, 150 mM HEPES, 0.01% DDM, 0.002% CHS, 10 μM LY341495, 300 μM VU0650786 (MedChemExpress)) for NAM-bound inactive mGlu3 sample. Peak fractions of mGlu3 dimer protein were collected and concentrated to 7~10 mg/ml for electron microscopy studies.

Cryo-EM grid preparation and data collection

For the preparation of cryo-EM grids, 3 μl purified mGlu3 sample was applied onto a freshly plasma-cleaned holey carbon grid (GryoMatrix-M024, R1.2/1.3, 300 mesh, Au), blotted for 7 s at 100% humidity with a Vitrobot Mark IV (ThermoFisher Scientific) and plunge frozen into liquid ethane cooled by liquid nitrogen. The first dataset of agonist-mGlu3 was collected on a Titan Krios at 300 kV accelerating voltage in the Center of Cryo-Electron Microscopy, Zhejiang University (Hangzhou, China). Micrographs were recorded using a Gatan K2 Summit direct electron detector in counting mode with magnification of 29,000×, corresponding to a pixel size of 1.014 Å. The total dose of 57.6 e-/Å2 was fractionated to 36 frames with 0.2 s per frame. Nominal defocus values ranged from -1.5 to -1.8 μm. The second dataset of antagonist-

NAM mGlu3 and the third dataset of antagonist-mGlu3 collected on a Titan Krios at 300 kV accelerating voltage in the Center of Cryo-Electron Microscopy, University of Science and Technology of China (Hefei, China). Micrographs were recorded using a Gatan K2 Summit direct electron detector in counting mode with magnification of 29,000×, corresponding to a pixel size of 1.01 Å. The total dose of 57.6 e-/Å2 was fractionated to 36 frames with 0.16 s per frame. Nominal defocus values ranged from -1.5 to -1.8 μm. The first dataset of agonist-mGlu3 included 11580 micrographs, the second dataset of NAM-bound inactive mGlu3 included 13106 micrographs and the third dataset of antagonist-mGlu3 included 4289 micrographs.

Imaging processing and 3D reconstruction

Three datasets were processed in cryoSPARC v2.15.0[1]. Movies were motion-corrected with built-in patch motion correction and contrast transfer function (CTF) estimation was performed with patch CTF estimation. Following CTF estimation, ~1000 particles were manually picked to generate 2D reference for auto-picking. For the first dataset of agonist-mGlu3, Auto-picking particles were extracted by four-times downscaling resulting in the pixel size of 4.056 Å. After three rounds of 2D classification, 862519 particles from well-defined 2D averages were extracted with a pixel size of 2.028 Å for further ab-initio reconstruction and heterogeneous refinement. A selected subset of particles from heterogeneous refinement were extracted with a pixel size of 1.014 Å and followed by C2 symmetry imposed in homogeneous refinement yielded a density map with nominal resolution of 3.82 Å according to the Fourier shell correlation (FSC) = 0.143 gold standard criterion. Followed heterogeneous refinement and Non-uniform

refinement then improved the resolution to 3.68 Å. The second dataset of NAM-bound inactive mGlu3 was similarly processed in cryoSPARC. With a selected subset of particles yielded a 3.93 Å a density map from homogeneous refinement. Followed heterogeneous refinement and non-uniform refinement then improved the resolution to 3.71 Å. The third dataset of NAM-bound inactive mGlu3 was similarly processed in cryoSPARC. With a selected subset of particles yielded a 4.35 Å a density map from homogeneous refinement. Followed heterogeneous refinement and Non-uniform refinement then improved the resolution to 4.17 Å.

Model building

The initial model for the human mGlu3 receptor was derived from mGlu5 (PDB code: 6N51 and 6N52) followed by extensive remodeling using COOT[2]. The N-terminal residues 1-30, residues 118-140, ICL2 residues 669-687 and C-terminal residues 824-879 of mGlu3 were not built due to the lack of corresponding densities. Structure refinement and model validation were performed using phenix.real_space_refine module in PHENIX[3]. The final model was subjected to refinement and validation in PHENIX. Figures were prepared using UCSF Chimera[4] or UCSF Chimera X[5].

CAMYEL biosensor assay for cAMP

The cAMP accumulation was measured by using the CAMYEL biosensor as previously described[6]. HEK-293 cells (ATCC, CRL-1573, lot: 3449904) were suspended and transfected using Lipofectamine 2000 with the appropriate expression constructs (for receptor: 30 ng - 60 ng optimized to make the same cell surface expression; EAAC 20 ng; CAMYEL 60 ng) per well in 96-well plate following the manufacturer’s protocols.

The ratio of DNA to Lipofectamine 2000 was 1:2. After a 24 h culture in 96-well plates, the cells were incubated in Glutamax medium for 1h and then in Cl^- free buffer (containing 146 mM Na D-gluconate, 4.2 mM K D-gluconate, 0.5 mM Mg D-gluconate, 10 mM HEPES and 1g/L glucose, pH 7.4) for 1h before the BRET measurements. For agonist-mediated cAMP inhibition (inhibition of cAMP), cells were pretreated for 5 min with 5 μM coelenterazine and 5 μM forskolin, and then stimulated for 5 min with different dose of LY2794193. For NAM inhibition of agonist-mediated cAMP response (increase of cAMP), cells were pretreated for 5 min with 5 μM coelenterazine h, 5 μM forskolin and 0.1 μM LY2794193, and then stimulated for 5 min with different dose of VU0650786. For the drug effect on the basal mGlu3 activity (increase of cAMP), cells were pretreated for 5 min with 5 μM coelenterazine h and then stimulated for 5 min with different dose of LY341495 or VU0650786 or LY341495 and VU0650786. The BRET signal was determined by calculating the ratio of the light emitted by Venus (535/30 nm) over that emitted by Rluc8 (475/30 nm) ($BRET^1$) using a Mithras LB 940 multimode microplate reader (Berthold Technologies, Bad Wildbad, Germany). The net BRET values were obtained by subtracting the background ratio from drug untreated cells. BRET changes were expressed as a percentage of the maximum response of the WT receptor for each mutant. Cell surface expression of the receptor subunits was detected by ELISA in parallel using anti-Flag or anti-HA antibodies coupled with horseradish peroxidase (Sigma-Aldrich).

Statistical analysis

Statistical analyses were performed on at least three individual data sets analyzed by

Graphpad prism. Data are means ± SEM from at least three independent experiments performed in technical triplicate. For dose-response experiments, data were normalized and analyzed using nonlinear curve fitting for the log (agonist) versus response (three parameters) curves.

Data availability

The cryo-EM density map and corresponding atomic coordinate of the LY341495-bound, LY341495 and VU0650786-bound, LY2794193-bound mGlu3 have been deposited in the Electron Microscopy Data Bank under the accession codes of EMD-32527, EMD-32526 and EMD-32530, respectively, and in the Protein Data Bank under accession codes 7WI8, 7WI6 and 7WIH, respectively. All data analyzed in this study are included in this paper and its Supplementary Information.

Reference:

1 Punjani, A., Rubinstein, J. L., Fleet, D. J. & Brubaker, M. A. cryoSPARC: algorithms for rapid unsupervised cryo-EM structure determination. *Nature methods* **14**, 290-296, doi:10.1038/nmeth.4169 (2017).

2 Emsley, P. & Cowtan, K. Coot: model-building tools for molecular graphics. *Acta Crystallogr D Biol Crystallogr* **60**, 2126-2132, doi:10.1107/S0907444904019158 (2004).

3 Afonine, P. V. *et al.* Real-space refinement in PHENIX for cryo-EM and crystallography. *Acta Crystallogr D Struct Biol* **74**, 531-544, doi:10.1107/S2059798318006551 (2018).

4 Pettersen, E. F. *et al.* UCSF Chimera--a visualization system for exploratory research and analysis. *Journal of computational chemistry* **25**, 1605-1612 (2004).

5 Goddard, T. D. *et al.* UCSF ChimeraX: Meeting modern challenges in visualization and analysis. *Protein science : a publication of the Protein Society* **27**, 14-25, doi:10.1002/pro.3235 (2018).

6 Jiang, L. I. *et al.* Use of a cAMP BRET sensor to characterize a novel regulation of cAMP by the sphingosine 1-phosphate/G13 pathway. *The Journal of biological chemistry* **282**, 10576-10584 (2007).

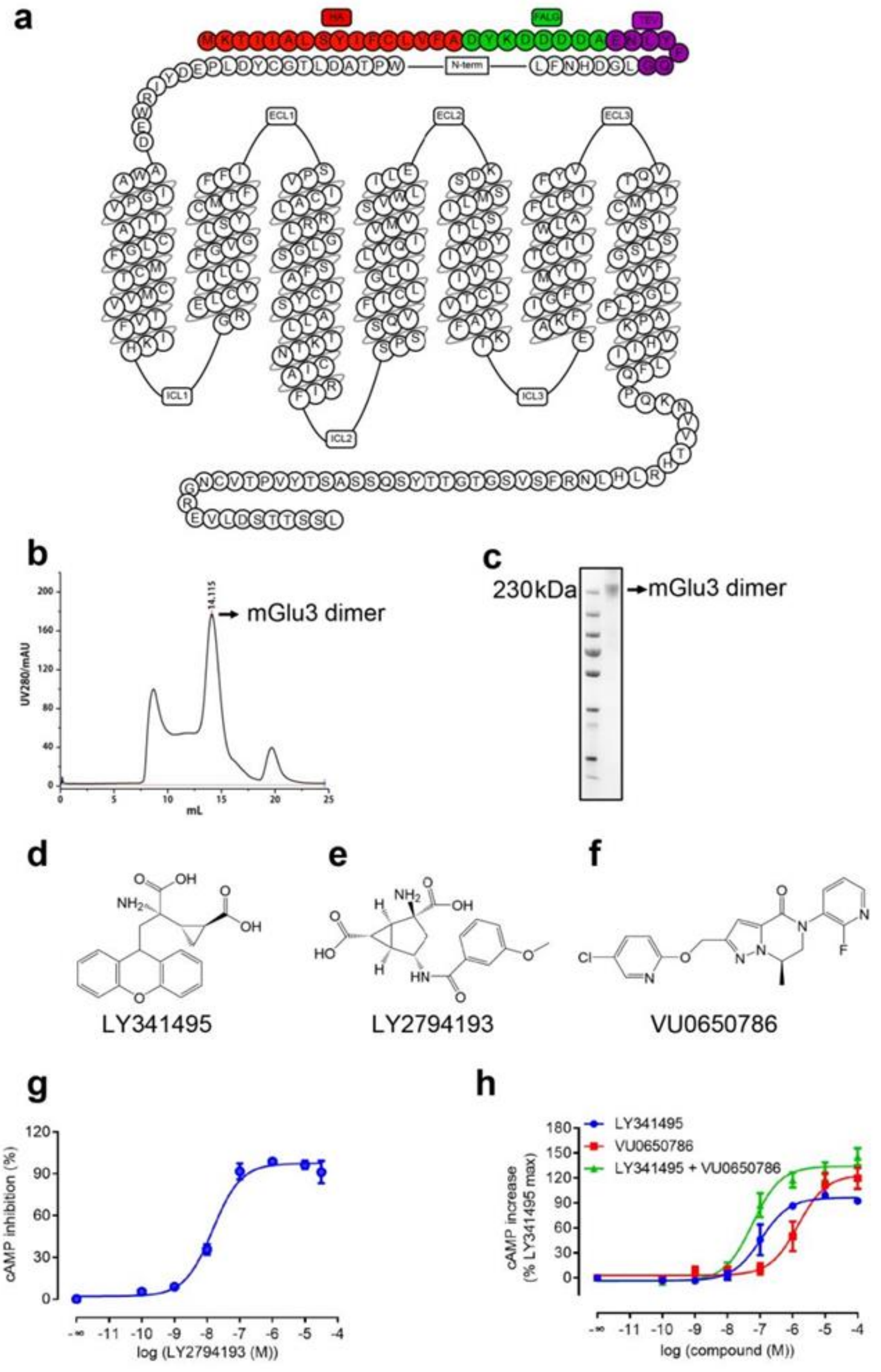

Fig. S1 Purification and characterization of full-length mGlu3.

a, Schematic diagram of mGlu3 used in the study. HA signal peptide (red), flag epitope (green) and TEV were linked at N-terminus. **b,** Size-exclusion chromatography profile of mGlu3. **c.** SDS-PAGE analysis of full-length mGlu3 dimer. **d-f,** The chemical structures of antagonist LY341495, agonist LY2794193 and NAM VU0650786. **g,** Agonist LY2794193 induced cAMP inhibition of mGlu3 measured by cAMP EPAC BRET sensor. **h,** cAMP EPAC BRET analysis revealed that antagonist (LY341495) and NAM (VU0650786) binding to mGlu3 can induce cAMP increase measured by cAMP EPAC BRET sensor. Data are means ± SEM from at least three independent experiments performed in technical triplicate in (**g**) and (**h**).

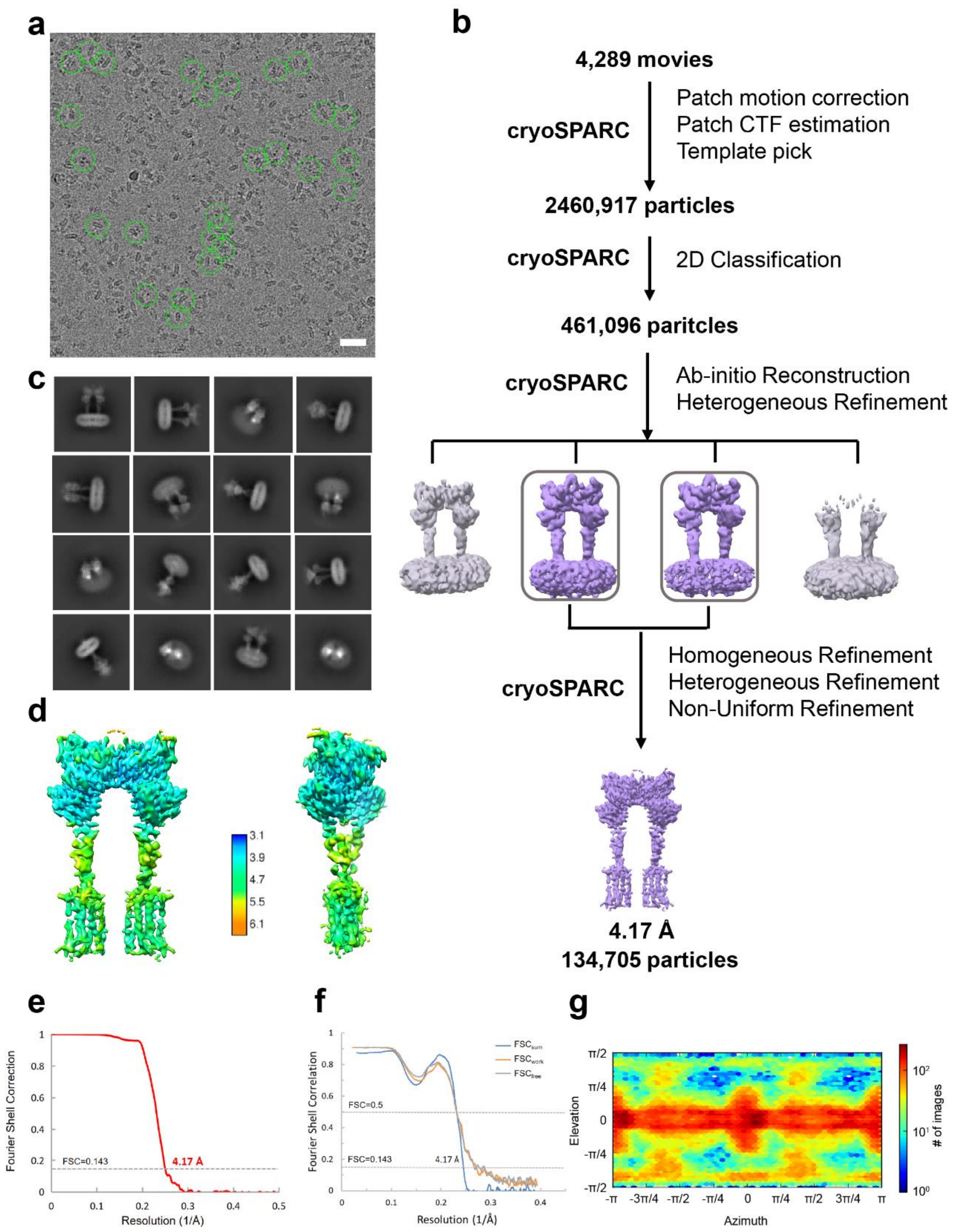

Fig. S2 Cryo-EM data processing of LY341495-bound mGlu3.

a, Representative micrograph after motion correction and dose weighting. **b,** Flow chart of cryo-EM data processing using cryoSPARC. **c**, 2D class averages of mGlu3 bound with LY341495. **d,** Density map of mGlu3 colored by local resolution estimation. **e,** Fourier shell correlation (FSC) curves for the mGlu3 LY341495-bound state cryo-EM maps of the global refinement from cryoSPARC. **f,** FSCsum/FSCwork/FSCfree validation curves of LY341495-bound mGlu3. **g,** Angular distribution of particles from the final cryo-EM reconstruction map.

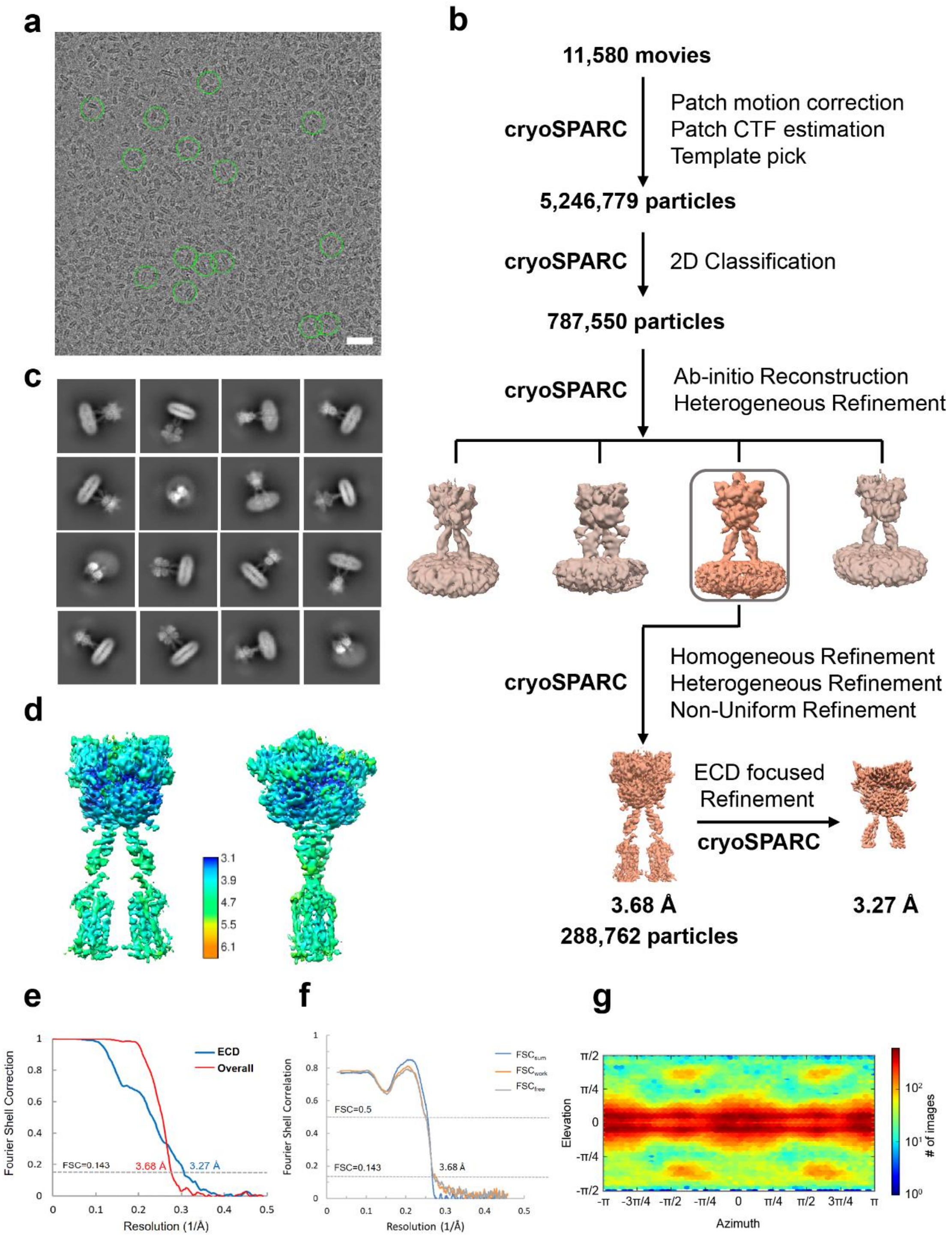

Fig. S3 Cryo-EM data processing of LY2794193-bound mGlu3.

a, Representative micrograph after motion correction and dose weighting. **b,** Flow chart of cryo-EM data processing using cryoSPARC. **c,** 2D class averages of mGlu3 bound with LY2794193. **d,** Density map of mGlu3 colored by local resolution estimation. **e,** Fourier shell correlation (FSC) curves for the mGlu3 LY2794193-bound state cryo-EM maps of the ECD focused refinement and the global refinement from cryoSPARC. **f,** FSCsum/FSCwork/FSCfree validation curves of LY2794193-bound mGlu3. **g,** Angular distribution of particles from the final cryo-EM reconstruction map.

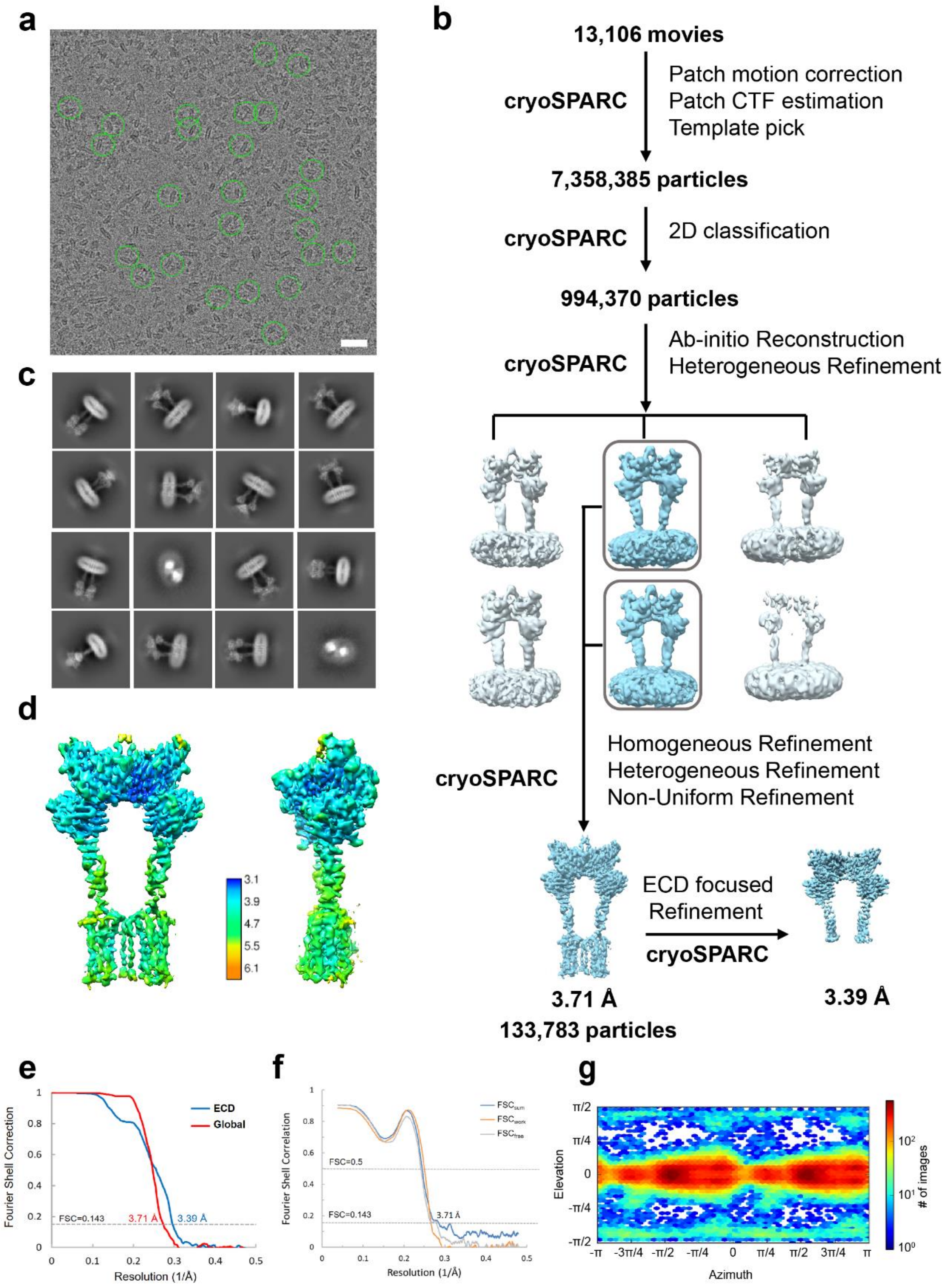

Fig. S4 Cryo-EM data processing of LY341495/ VU0650786-bound mGlu3.

a, Representative micrograph after motion correction and dose weighting. **b,** Flow chart of cryo-EM data processing using cryoSPARC. **c,** 2D class averages of mGlu3 bound with LY341495 and VU0650786. **d,** Density map of mGlu3 colored by local resolution estimation. **e,** Fourier shell correlation (FSC) curves for the mGlu3 LY341495/ VU0650786-bound state cryo-EM maps of the ECD focused refinement and the global refinement from cryoSPARC. **f,** FSCsum/FSCwork/FSCfree validation curves of LY341495/VU0650786-bound mGlu3. **g,** Angular distribution of particles from the final cryo-EM reconstruction map.

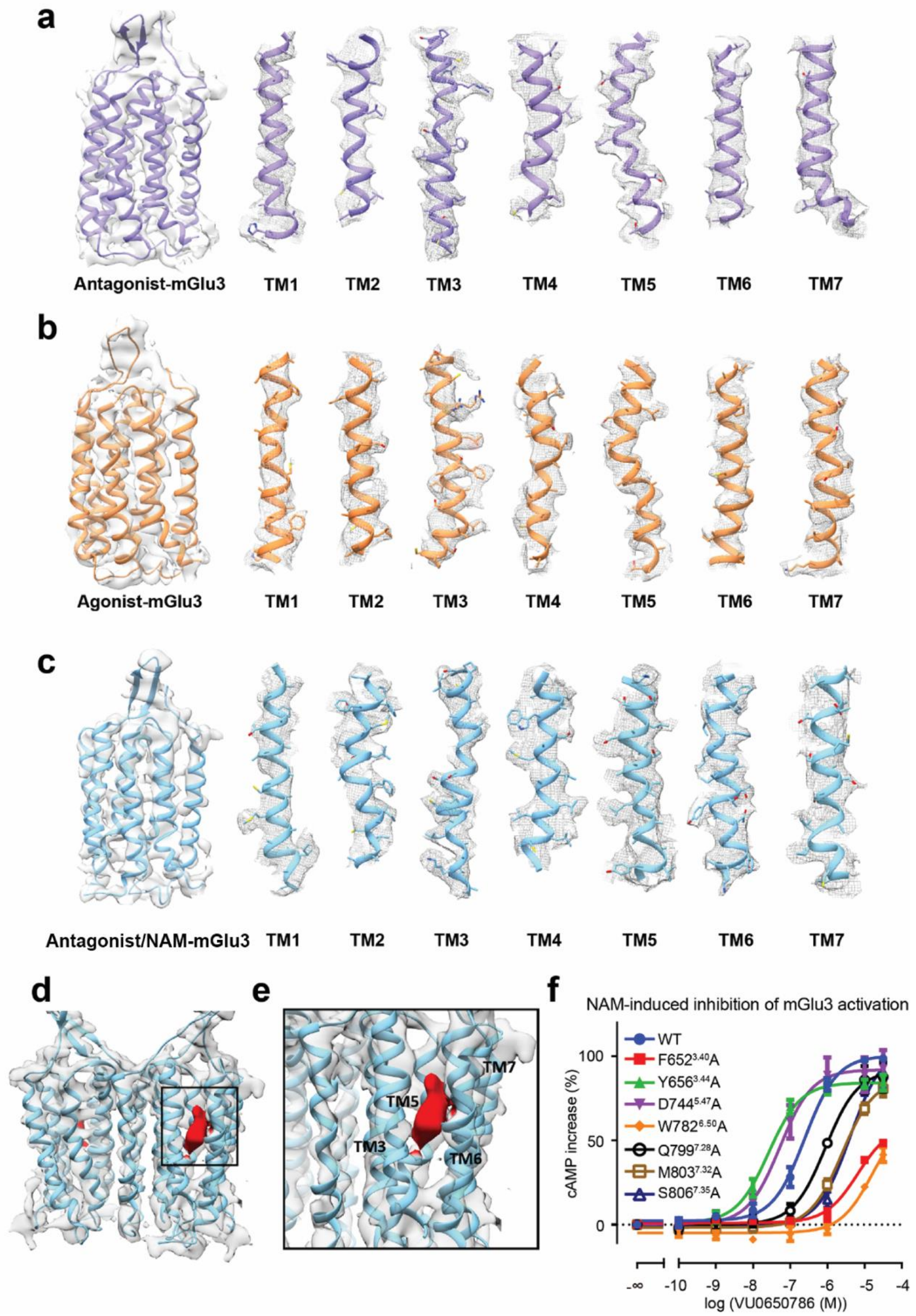

Fig. S5 Cryo-EM densities of representative segments of mGlu3.

a-c, Representative cryo-EM densities and fitted atomic models of TMD and transmembrane helix for antagonist-bound (**a**), agonist-bound (**b**) and antagonist/NAM-bound (**c**) mGlu3. **d,** EM density and model of the TMDs for antagonist/NAM-bound mGlu3 with additional map(red) corresponding to negative allosteric modulator

VU0650786. **e,** Magnified view of the density of NAM VU0650786 inside the allosteric pocket formed by TM3, TM5, TM6 and TM7. **f,** VU0650786 concentration response for WT and mutants designed in NAM binding pocket measured by cAMP EPAC BRET sensor in presence of LY2794193 (0.1 μM LY2794193). Data are means ± SEM from at least three independent experiments performed in technical triplicate.

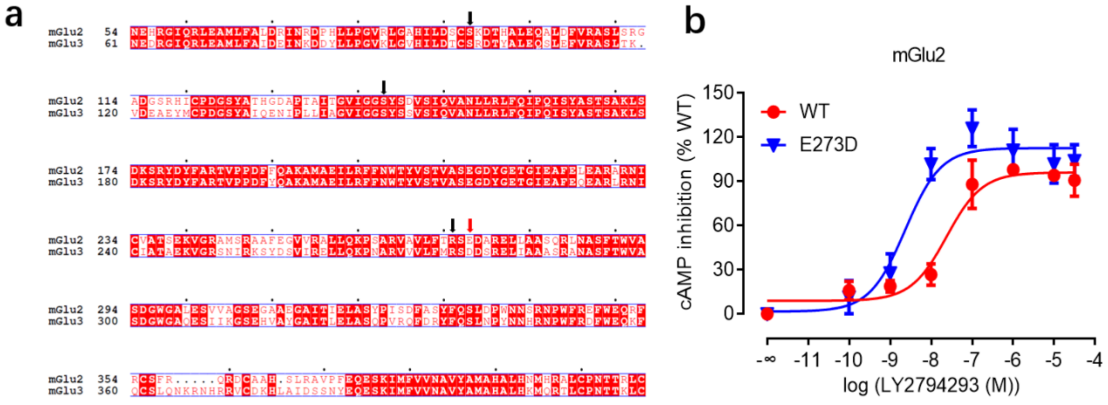

Fig. S6 Selectivity of agonist LY2794193 to mGlu3.

a, Sequence alignment of othosteric binding pocket of mGlu2 and mGlu3. The residues of mGlu3 that formed interaction with the m-methoxyphenyl ring of LY2794193 were indicated with arrows and the mismatch residue D279 of mGlu3 corresponding to E273 of mGlu2 was indicated with red arrow. **b,** LY2794193 induced cAMP inhibition in mGlu2 WT and E273D mutant. Data are means ± SEM from at least three independent experiments performed in technical triplicate.

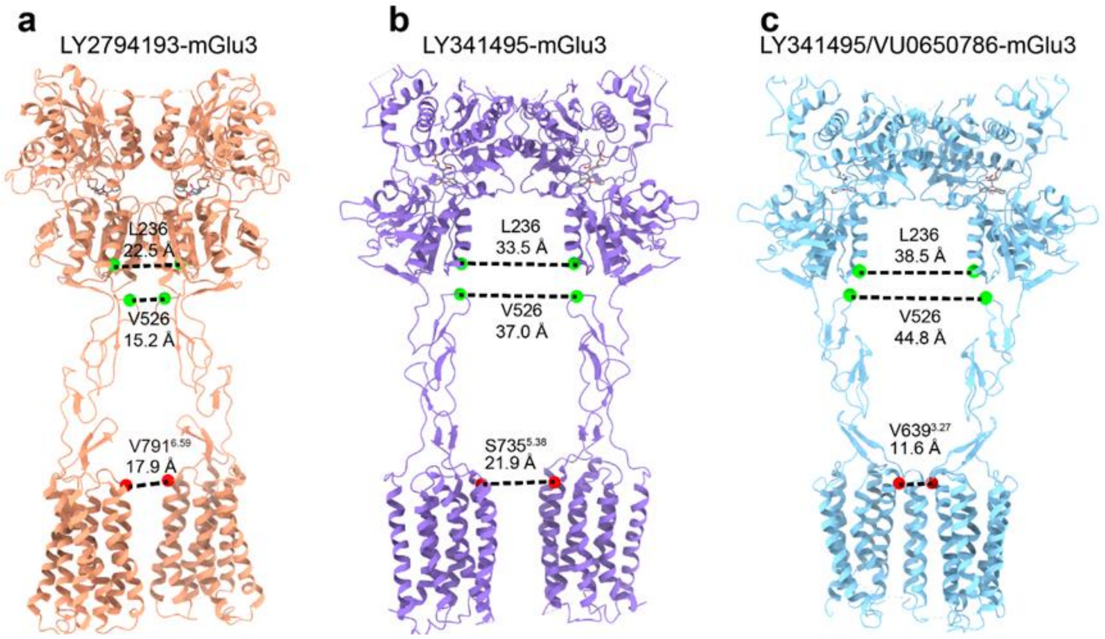

Fig. S7 Structural changes at the dimer interfaces during the activation of mGlu3.

a-c, The ribbon diagram representation of mGlu3 in the agonist-bound state(**a**), antagonist-bound state(**b**) and antagonist/NAM -bound state(**c**). The distances between different region of mGlu3 dimer were shown with dotted lines.

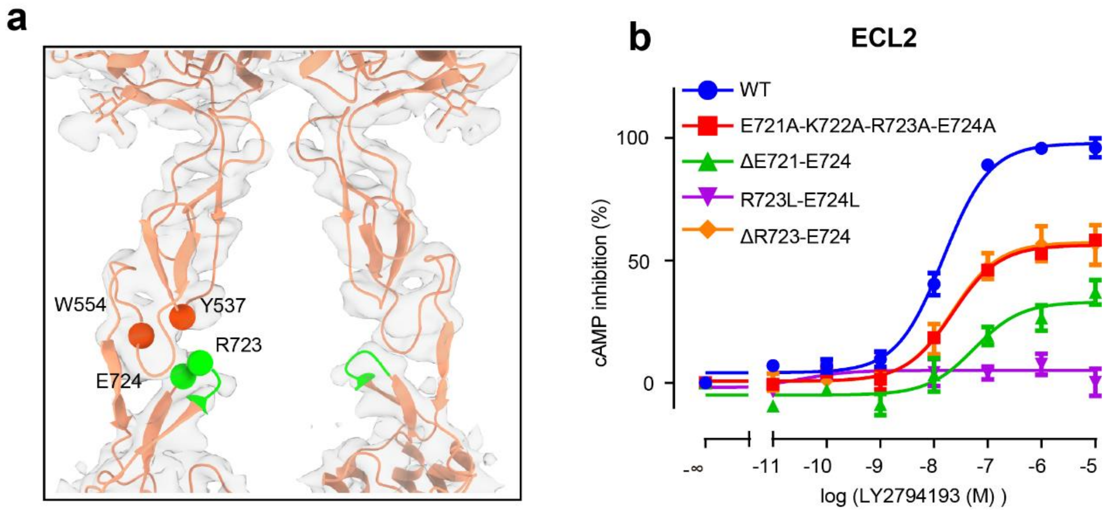

Fig. S8 ECL2 is necessary for activation by orthosteric agonists.

a, Map and model of the CRD and ECL2 interface. b, f, Mutations or deletions of residues in mGlu3 ECL2 impaired the agonist-induced cAMP inhibition. Data are means ± SEM from at least three independent experiments performed in technical triplicate.

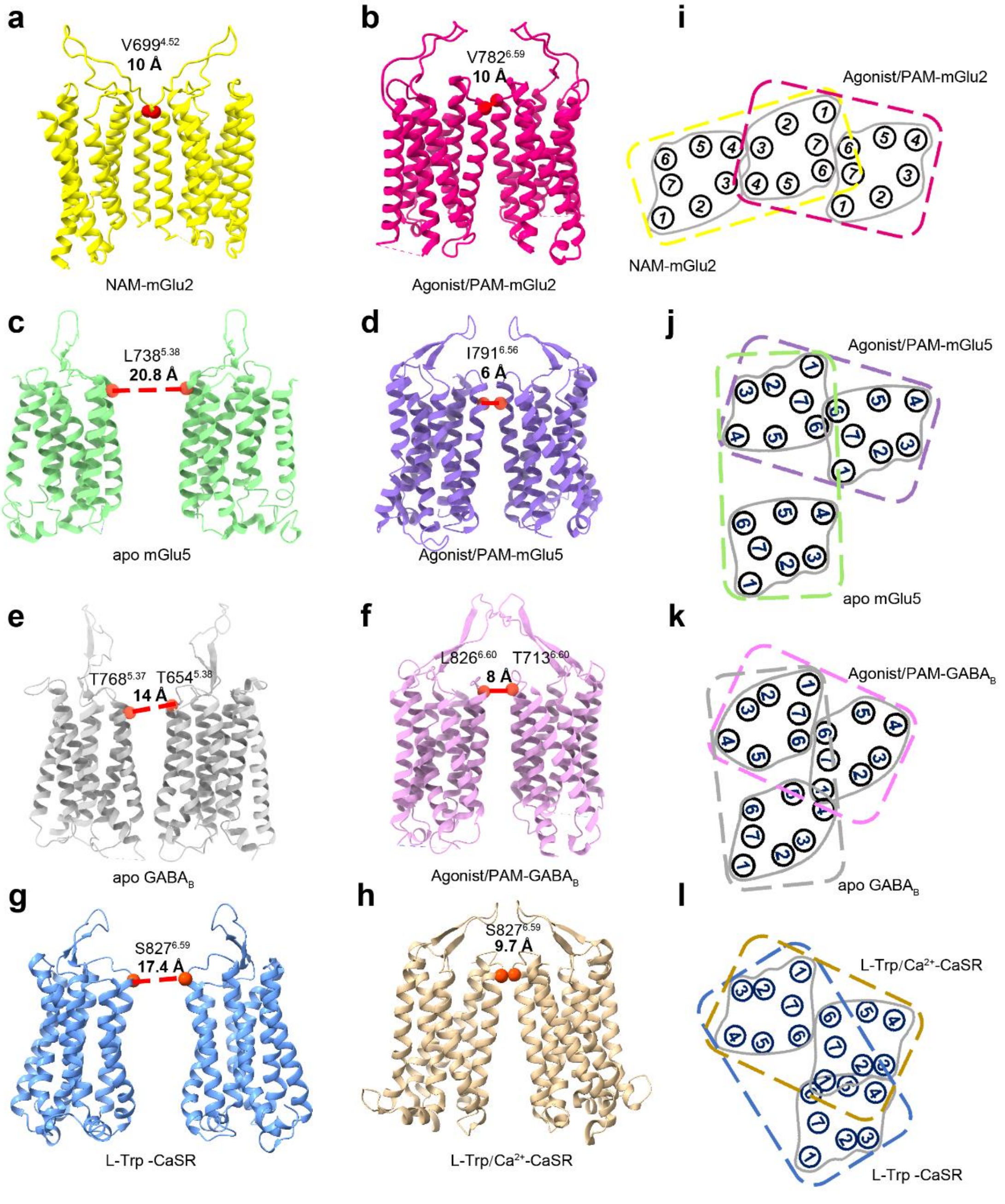

Fig. S9 Comparison of TMD of mGlu2, mGlu5, GABA$_B$ and CaSR.

a, b, Side views of the TMDs in NAM-bound state (**a,** PDB: 7EPA) and agonist/PAM-bound state (**b,** PDB: 7EPB) of mGlu2. **c, d,** Side views of the TMDs in apo state (**c,** PDB: 6N52) and agonist/PAM-bound state (**d,** PDB: 6N51) of mGlu5. **e, f,** Side views of TMDs in apo state (**e,** PDB: 6VJM) and agonist/PAM-bound active state (**f,** PDB: 6UO8) of GABA$_B$. **g, h,** Side views of TMDs in L-Trp (**g,** PDB: 7DTU) and L-Trp/Ca^{2+} (**h,** PDB: 7DTV) bound state of CaSR. The red dotted lines indicate the proximate distances between the two TMD monomers in **a-h**. **i-l,** The TMD orientation of mGlu2 (**i**), mGlu5 (**j**), GABA$_B$ (**k**) and CaSR (**l**) during activation in extracellular view.

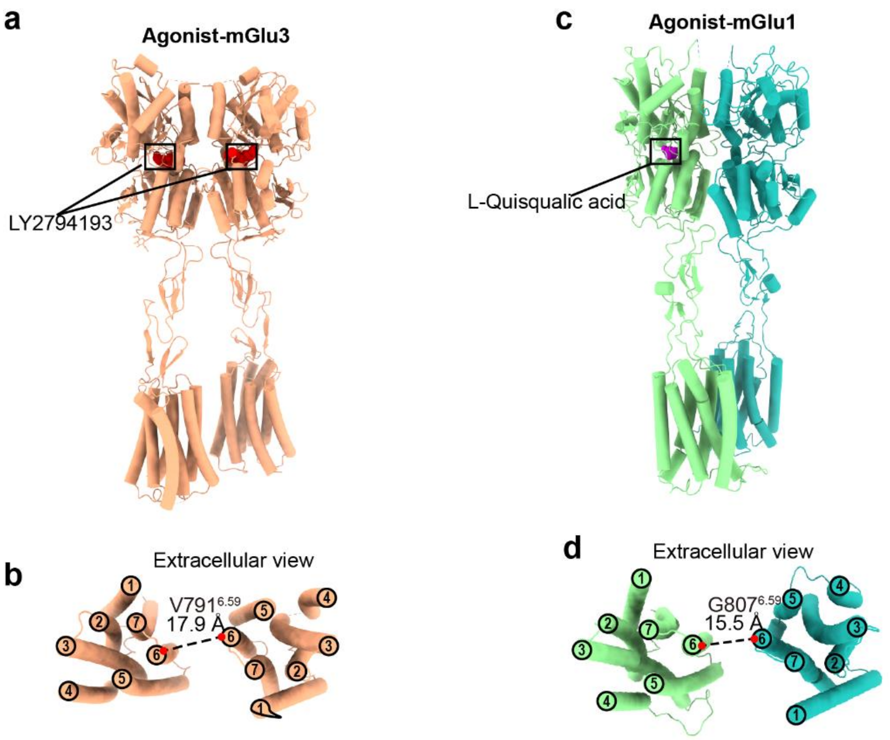

Fig. S10 Structural comparison of agonist-bound mGlu3 and agonist-bound mGlu1.

a, Model of agonist-bound mGlu3 with both VFT domain bound with agonist LY2794193. **b,** Extracellular view of agonist-bound mGlu3. The distances of TMD dimer were indicated with dotted lines. **c,** Model of agonist-bound mGlu1(PDB: 7DGE) with both VFT domain bound with agonist L-Quisqualic acid**. d,** Extracellular view of agonist-bound mGlu1(PDB: 7DGE). The distances of TMD dimer were indicated with dotted lines.

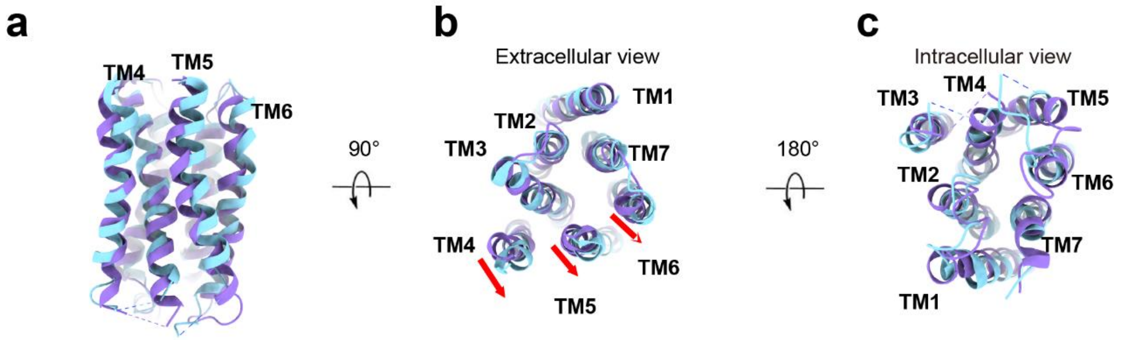

Fig. S11 Comparison of the mGlu3 TMD in antagonist-bound state and antagonist/NAM-bound state.

a, b, c, Side (**a**), extracellular (**b**), and intracellular views (**c**) of the superposed TMD of the antagonist-bound state (purple) and the antagonist/NAM-bound state (blue) of mGlu3. The red arrows indicate the shift of the transmembrane helix after NAM binding.

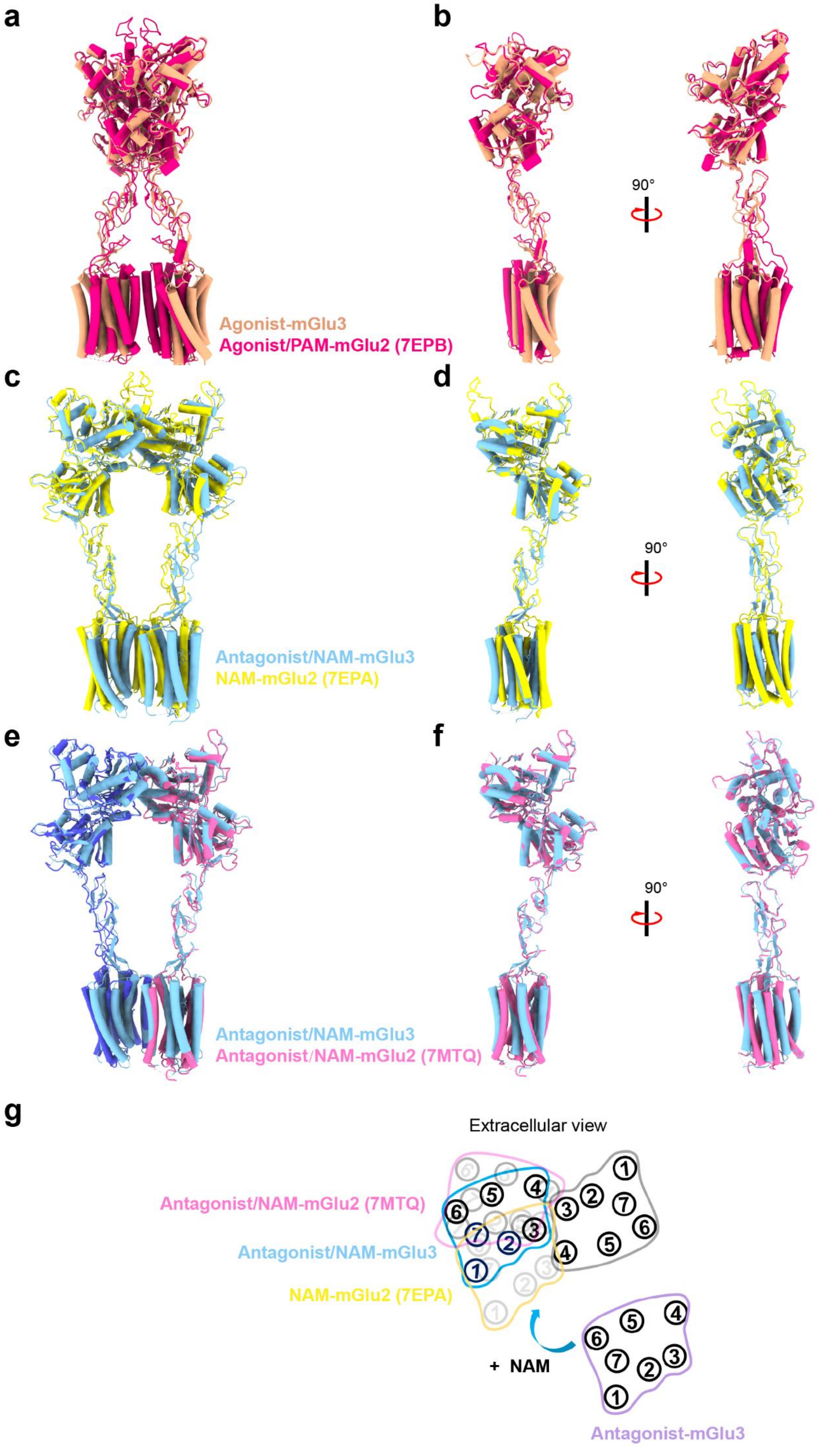

a
b
90°
Agonist-mGlu3
Agonist/PAM-mGlu2 (7EPB)
c
d
90°
Antagonist/NAM-mGlu3
NAM-mGlu2 (7EPA)
e
f
90°
Antagonist/NAM-mGlu3
Antagonist/NAM-mGlu2 (7MTQ)
g
Extracellular view
Antagonist/NAM-mGlu2 (7MTQ)
Antagonist/NAM-mGlu3
NAM-mGlu2 (7EPA)
+ NAM
Antagonist-mGlu3

Fig. S12 Structural comparison of Group II mGluRs.

a, b, Structural comparison of mGlu2 in the agonist/PAM bound state (red, PDB: 7EPB) with mGlu3 (orange) in the agonist bound state based on dimer superposition (**a**) and monomer superposition (**b**). **c, d, S**tructural comparison of mGlu2 (yellow, PDB: 7EPA) in the NAM bound state with mGlu3 (blue) in the antagonist/NAM-bound state based on dimer superposition (**c**) and monomer superposition (**d**). **e, f, S**tructural comparison of mGlu2 (pink, PDB: 7TMQ) in the antagonist/NAM-bound state with mGlu3 in the antagonist/NAM-bound state based on dimer superposition (**e**) and monomer superposition (**f**). **g**, Schematic diagram of the extracellular view of TMDs orientations in antagonist/NAM bound state mGlu3(blue), antagonist bound mGlu3(purple), antagonist/NAM-bound mGlu2 (PDB: 7MTQ) and NAM bound mGlu2(PDB: 7PEA).

Table S1 | Statistics of cryo-EM data collection, 3D reconstruction and model refinement.

**Data Collection**			
**Protein**	mGlu3 - LY341495	mGlu3 - LY2794193	mGlu3 - LY341495/VU0650786
**Microscope**	FEI Titan Krios	FEI Titan Krios	FEI Titan Krios
**Voltage (kV)**	300	300	300
**Detector**	Gatan K2 Summit	Gatan K2 Summit	Gatan K2 Summit
**Detector mode**	Counting	Counting	Counting
**Pixel size (Å)**	1.01	1.014	1.01
**Defocus range (µm)**	-1.5 ~ -1.8	-1.5 ~ -1.8	-1.5 ~ -1.8
**Electron dose (e^-/Å^2)**	57.6	57.6	57.6
**Frames per image**	36	36	36
**Exposure time (s)**	5.76	7.2	5.76
**Number of images**	4,289	11580	13106
**3D reconstruction**			
**Software**	cyroSPARC v2.15.0	cyroSPARC v2.15.0	cyroSPARC v2.15.0
**Particle number**	134,705	288762	133783
**Symmetry**	C2	C2	C2
**Overall resolution (Å)**	4.17	3.68	3.71
**Model refinement**			
**Model composition**			
**Chains**	2	2	2
**Ligands**	Z99:2 NAG:2	CWY:2 NAG:2	CWY:2 NAG:2 LIG:2
**Non-hydrogen atoms**	9,276	10,034	9,792
**Protein residues**	1,446	1,482	1,446
**Bonds (RMSD)**			
**Length (Å)**	0.007	0.004	0.006

**Angles (°)**	1.023	0.961	0.985
**Ramachandran plot (%)**			
**Outliers**	0.00	0.14	0.14
**Allowed**	13.22	10.26	12.41
**Favored**	86.78	89.60	87.45
**Rotamer outliers (%)**	0.91	1.20	1.27
**MolProbity score**	1.92	1.95	1.93
**Clash score**	5.51	5.93	4.64

Table S2 | LY2794193 or VU0650786-induced cAMP change of wild-type and mutant mGlu3.

		LY2794193		Mean ± SEM (n)		
		EC_{50} (nM)	Fold shift	pEC_{50}	E_{max}	Expression
LY2794193 binding pocket	WT	13.5	1.0	7.9 ± 0.1 (21)	100 (21)	100 (21)
	Y150A	86.4	6.4	7.1 ± 0.0 (3)	91.0 ± 3.7 (3)	105.0 ± 11.6 (3)
	R277A	3.5	0.3	8.5 ± 0.1 (4)	79.5 ± 8.2 (4)	96.2 ± 6.3 (4)
	S278A	17.4	1.3	7.8 ± 0.1 (4)	95.3 ± 9.3 (4)	96.1 ± 8.7 (4)
	D279E	207.2	15.4	6.7 ± 0.1 (3)	103.1 ± 5.5 (3)	105.4 ± 6.2 (3)
ECL2	ΔE721-E724	49.6	3.7	7.3 ± 0.3 (3)	37.0 ± 5.0 (3)	138.8 ± 21.5 (3)
	E721A-K722A-R723A-E724A	21.7	1.6	7.7 ± 0.1 (3)	58.2 ± 2.1 (3)	113.3 ± 5.6 (3)
	R723L-E724L	nd	nd	nd	nd	79.0 ± 1.1 (3)
	ΔR723-E734	20.7	1.5	7.7 ± 0.2 (3)	57.5 ± 7.6 (3)	96.5 ± 6.4 (3)
VU0650786 binding pocket	F652A	37.3	2.8	7.4 ± 0.1 (3)	80.7 ± 5.8 (3)	126.0 ± 0.9 (3)
	Y656A	13.1	1.0	7.9 ± 0.1 (3)	93.7 ± 3.7 (3)	91.8 ± 3.6 (3)
	D744A	12.9	1.0	7.9 ± 0.3 (3)	104.2 ± 1.1 (3)	108.0 ± 13.9 (3)
	W782A	7.1	0.5	8.2 ± 0.1 (3)	96.4 ± 12.5 (3)	102.5 ± 4.0 (3)
	Q799A	6.8	0.5	8.2 ± 0.1 (3)	96.3 ± 10.6 (3)	97.7 ± 8.1 (3)
	M803A	33.3	2.5	7.5 ± 0.1 (3)	93.3 ± 9.8 (3)	105.0 ± 0.6 (3)
	S806A	21.0	1.6	7.7 ± 0.1 (3)	103.8 ± 11.3 (3)	100.4 ± 5.7 (3)

		VU0650786		Mean ± SEM (n)
		IC_{50} (nM)	Fold shift	pIC_{50}
VU0650786 binding pocket	WT	271.3	1.0	6.6 ± 0.1 (7)
	F652A	6299.9	23.2	5.2 ± 0.1 (3)
	Y656A	25.8	0.1	7.6 ± 0.1 (3)
	D744A	50.7	0.2	7.3 ± 0.2 (3)
	W782A	15703.6	57.9	4.8 ± 0.2 (3)
	Q799A	863.6	3.2	6.1 ± 0.0 (3)
	M803A	2475.5	9.1	5.6 ± 0.1 (3)
	S806A	4076.9	15.0	5.4 ± 0.1 (3)
